# A real‐world retrospective–prospective analysis of efficacy and safety of combined ixazomib, lenalidomide, and dexamethasone in relapsed/refractory multiple myeloma: The northern Italy experience

**DOI:** 10.1002/cam4.7071

**Published:** 2024-04-01

**Authors:** Anna Furlan, Michele Cea, Laura Pavan, Monica Galli, Cristina Clissa, Silvia Mangiacavalli, Anna Maria Cafro, Stefania Girlanda, Francesca Patriarca, Claudia Minotto, Giovanni Bertoldero, Gregorio Barilà, Anna Pascarella, Albana Lico, Rossella Paolini, Nicholas Rabassi, Norbert Pescosta, Marika Porrazzo, Giovanni De Sabbata, Alessandra Pompa, Giulia Bega, Stefania Cavallin, Francesca Guidotti, Magda Marcatti, Maurizio Rupolo, Angelo Belotti, Filippo Gherlinzoni, Renato Zambello

**Affiliations:** ^1^ Divisione di Ematologia Ospedale Ca' Foncello di Treviso, ASL 2 Treviso Italy; ^2^ Hematology Unit, Department of Internal Medicine (DiMI) University of Genoa, IRCSS Ospedale Policlinico San Martino Genova Italy; ^3^ Padua University School of Medicine Hematology and Clinical Immunology Padova Italy; ^4^ Hematology Division, Ospedale Papa Giovanni XXIII Bergamo Italy; ^5^ Hematology Unit and Stem Cells Transplant Center Azienda Ospedaliera Universitaria Integrata di Verona Verona Italy; ^6^ Hematology Division IRCCS Fondazione Policlinico San Matteo Pavia Italy; ^7^ Hematology Unit ASST GOM Niguarda Milano Italy; ^8^ Medical Oncology and Hematology Unit ASST Fatebenefratelli Sacco, PO Fatebenefratelli Milano Italy; ^9^ Hematology Unit Azienda Sanitaria Universitaria Friuli Centrale, DAME, Udine University School of Medicine Udine Italy; ^10^ Medical Oncology and Hematology Unit Azienda ULSS 3 Serenissima Mirano Italy; ^11^ Hematology Unit Azienda ULSS3 Serenissima, Ospedale dell'Angelo Venezia‐Mestre Italy; ^12^ Hematology Unit Azienda ULSS8 Berica, Ospedale San Bortolo Vicenza Italy; ^13^ Hematology Unit Ospedale Santa Maria della Misericordia Rovigo Italy; ^14^ Hematology Unit and Stem Cells Transplant Center Ospedale Provinciale Bolzano Bolzano Italy; ^15^ Hematology Unit Ospedale Maggiore Trieste Italy; ^16^ Hematology Unit IRCCS Fondazione Ca' Granda, Ospedale Maggiore Policlinico Milano Italy; ^17^ Medical Oncology Unit Ospedale G. Fracastoro, Azienda ULSS 9 Scaligera Verona Italy; ^18^ Medical Oncology Unit Ospedale di Vittorio Veneto, Azienda ULSS 2 Marca Trevigiana Vittorio Veneto Italy; ^19^ Division of Hematology, Department of Medicine Ospedale Valduce Como Italy; ^20^ Hematology Unit IRCSS Ospedale San Raffaele Milano Italy; ^21^ SOSD Oncoematologia Istituto Nazionale Tumori Aviano Aviano Italy; ^22^ Hematology Unit ASST Spedali Civili di Brescia Brescia Italy

**Keywords:** efficacy, ixazomib–lenalidomide–dexamethasone, lenalidomide‐exposed, lenalidomide‐refractory, real‐life, relapsed/refractory multiple myeloma, safety

## Abstract

**Introduction:**

Ixazomib, lenalidomide, and dexamethasone (IRd) have been approved for the treatment of relapsed/refractory multiple myeloma (RRMM) based on the results of the TOURMALINE‐MM1.

**Objectives and Methods:**

We conducted a retrospective–prospective analysis of 106 RRMM patients (pts) treated with IRd in 21 centers in Northern Italy, with the aim to evaluate the efficacy and safety of IRd in real life.

**Results:**

At IRd initiation, 34% of pts were aged ≥75 (median 72.5), 8.5% had an ECOG performance status ≥2, 54.7% of evaluable pts carried high‐risk cytogenetic abnormalities [del17p and/or t(4;14) and/or t(14;16) and/or 1 g gain/amp], 60.2% had received ≥2 prior lines of therapy (pLoT), 57.5% were lenalidomide (Len)‐exposed (including both Len‐sensitive and Len‐refractory pts), and 22% were Len‐refractory. Main G ≥3 adverse events (AEs) were thrombocytopenia (16%) and neutropenia (12.3%). G ≥3 non‐hematologic AEs included infections (9.4%) and GI toxicity (diarrhea 5.7%, hepatotoxicity 2.8%), VTE, skin rash, and peripheral neuropathy were mainly G1‐2. The overall response rate was 56.4% (≥VGPR 30%). With a median follow‐up of 38 m, median PFS (mPFS) was 16 m and the 1‐year OS rate was 73%. By subgroup analysis, an extended PFS was observed for pts achieving ≥VGPR (mPFS 21.2 m), time from diagnosis to IRd ≥5 years (26.2 m), 1 pLoT (34.4 m), Len‐naïve (NR), age ≥70 (20 m). In pts exposed to Len, non‐refractory in any prior line and immediately prior to IRd, mPFS was 16 and 18 m, respectively. An inferior PFS was seen in Len‐refractory pts (4.6 m). By multivariate analysis, independent predictors of PFS were age ≥70 (HR 0.6), time from diagnosis ≥5 years (HR 0.32), refractoriness to Len in any prior line (HR 3.33), and immediately prior (HR 4.31).

**Conclusion:**

IRd might be effective and safe in RRMM pts with an indolent disease, in early lines of treatment, and who proved Len‐sensitive, independent of age, and cytogenetic risk.

## 1 INTRODUCTION

The triplet combination of ixazomib, lenalidomide, and dexamethasone (IRd) has been approved for the treatment of relapsed/refractory multiple myeloma (RRMM) based on the results of the pivotal TOURMALINE‐MM1 trial comparing IRd and placebo‐Rd (lenalidomide and dexamethasone).^1^ The TOURMALINE‐MM1 reported superior median progression‐free survival (mPFS) with IRd (20.6 vs. 14.7 months) (m) and overall response rates (ORR) (78% vs. 72%) compared with placebo‐Rd. The corresponding rates of complete response plus very good partial response (CR+VGPR) were 48% and 39%, respectively.

In the routine clinical practice, the all‐oral route of administration and the favorable safety profile make this regimen potentially suitable for the treatment of elderly, frail patients (pts)^2^, ^3^, ^4^ who are under‐represented in the clinical studies.^5^, ^6^ Several observational studies^7^, ^8^, ^9^, ^10^, ^11^, ^12^, ^13^, ^14^, ^15^ of IRd have confirmed the effectiveness of IRd with ORR ranging from 60% to 74% and mPFS from 11.4 to 27.6 m in real‐world setting, despite a higher prevalence of elderly and heavily pre‐treated pts, compromised performance status (PS), and advanced stage disease. A proportion of pts ranging from 17% to 38% in the published real‐world studies had previous exposure to lenalidomide (Len) compared with only 12% in the MM1 study. Len‐refractory pts were excluded from the pivotal trial and are very poorly represented in the real‐world population included in the observational studies published to date. However, over the last few years, an increasing proportion of MM pts, both transplant eligible and non‐transplant eligible, receive Len‐based first line regimens until progression, with the result that the vast majority are Len‐exposed and, more often, Len‐refractory as early as at first relapse. This raises the issue of the feasibility of this Len‐based treatment in previously Len‐exposed or refractory pts and the optimal placing within the current therapeutic scenario.^16^, ^17^

In this perspective, we conducted an observational analysis of 106 RRMM pts treated with IRd between January 2017 and May 2021 in 21 centers in Northern Italy, with the aim to evaluate efficacy and safety of IRd in a real‐world population including a significant proportion of Len‐refractory pts (20.7%) in addition to Len‐exposed, non‐refractory pts (36.8%). The issue of rechallenge with this all‐oral, Len‐based, triplet regimen, might be especially relevant for the elderly population, where frailty and comorbidities limit the therapeutic options in the relapsed/refractory setting.

## 2 METHODS

### 2.1 PATIENTS

One hundred six RRMM pts starting IRd in routine care between January 2017 and May 2021 in 21 Northern Italy Centers were enrolled in a retrospective/prospective observational study. All pts consented to the processing of personal data. The study comprises a retrospective phase (chart review of the period from IRd initiation to enrolment) followed by a 24‐month prospective follow‐up period (cut‐off date: May 31, 2023). Inclusion criteria include diagnosis of RRMM; age ≥18 years; all subjects must *have* received at *least 2* consecutive *cycles* of IRd treatment until May 31, 2021, and have evaluable response data; written informed consent must be obtained. Exclusion criteria *include lack* of informed consent for participation.

Twenty‐six pts were analyzed only retrospectively; the remaining 80 pts were analyzed both retrospectively and prospectively. The median follow‐up was 38 m.

### 2.2 Study design

All patients received oral ixazomib (Ixa) (4 mg on Days 1, 8, and 15) in association with dexamethasone (Dex) 20 mg on Days 1, 8, and 15 and lenalidomide (Len) 25 mg orally on Days 1–21 of each 28‐day cycle. The dose of each drug was adjusted, according to drug recommendations, in case of specific pre‐existing comorbidities and during treatment based on intervening toxicities.

Disease response was based on investigator assessment according to the International Myeloma Working Group (IMWG) consensus criteria.^18^ The safety assessment was based on reports of hematological and non‐hematological toxicities, including anemia, neutropenia, thrombocytopenia, infections, venous thromboembolism (VTE), skin rash, increase in liver function tests, and gastrointestinal (GI) and neurological toxicity. All adverse events (AEs) were recorded using the Common Terminology Criteria for Adverse Events (CTCAEs) version 5.0. Renal insufficiency (RI) was defined as estimated glomerular filtration rate (eGFR) ≤60 mL/min.

The primary endpoint was to evaluate the safety and efficacy of IRd treatment, in terms of toxicity, ORR, PFS and OS. Secondary endpoints were to evaluate the impact of age, depth of response, cytogenetic risk, ISS, RI, number of prior lines of therapy (pLoT), time from diagnosis to IRd, exposure and refractoriness to Len, time interval between the last Len administration and IRd, and starting Len dose.

This study was conducted in accordance with the ethical principles of the Declaration of Helsinki.^19^

### 2.3 Statistical analysis

Response to therapy was assessed according to the IMWG criteria.^18^ ORR was calculated considering the achievement of at least a partial response (PR). Time to events endpoints was defined as the time from start of treatment to the relative event occurring. PFS was assessed depending on relapse/disease progression or death, whichever came first, while OS was calculated considering only deaths. The Kaplan–Meier method and relative survival curves were adopted to estimate PFS; comparisons between subgroups were evaluated by the Mantel–Cox log rank test. Semi‐parametric Cox regression univariate analysis was performed aiming to identify prognostic factors affecting PFS. To confirm whether those prognostic factors maintained an independent role, statistically significant variables (a *p* value <0.05 was considered to be statistically significant) were subsequently included in a multivariable Cox model adjusted for age. Analyses were performed using R (version 4.1.3).^20^ All estimates subject to inference were reported together with their 95% confidence intervals (CI) and 5% was chosen for all analyses as the max alpha error admitted and considered for statistical significance.

## 3 RESULTS

### 3.1 Patient characteristics

One hundred six pts, who received at least two cycles of IRd during the observation period, were retrospectively and prospectively evaluated. The reason for starting the IRd regimen was symptomatic progressive disease (PD) according to CRAB criteria in 47% of pts, and biochemical relapse in 53%.^21^ Patient characteristics at study entry are summarized in Table 1. At IRd initiation, the median age was 72.5, with 34% of pts aged ≥75. 8.5% had an ECOG performance status (PS) ≥2, 54.7% of evaluable pts (41 out of 75) carried HRCA [del17p and/or t(4;14) and/or t(14;16) and/or 1 g gain/amp], detected by fluorescence in situ hybridization (FISH); 25.3% of evaluable pts (19 out of 75) had del17p; RI, defined as eGFR ≤60 mL/min, was present in 31.6%, being severe (eGFR <30 mL/min) in only 3 pts (3.8%). 39.8% had ISS stage III. The median number of prior lines of therapy (pLoT) was 2 (range 1–7) with the majority of pts (60.2%) receiving ≥2. In more detail, 39.8% and 21.7% of pts received IRd at first and second relapse, respectively, while 38.5% had received 3 or more pLoT. 57.5% were exposed to Len (including both Len‐sensitive and Len‐refractory pts), 22% were Len‐refractory. The great majority of pts (78.3%) had received prior bortezomib (13.2% of them were refractory), and 42% underwent a prior autologous stem cell transplant (ASCT). Median time from diagnosis to IRd was 62 m.

**TABLE 1:** Patient characteristics at IRd initiation compared with the study population in the Tourmaline‐MM1 trial.

Characteristics	Study population (*n* = 106)	Tourmaline‐MM1 (*n* = 722)
Sex (*n*, %)		
Male	46 (43.4)	409 (57)
Female	60 (56.6)	313 (43)
Age (*n*, %)		
Median	72.5	66
≥75	36 (34)	108 (15)
<75	70 (66)	614 (85)
ECOG (*n*, %)		
≥2	9 (8.5)	42/712 (6)
ISS (*n*, %)		
I	26 (24.7)	459 (64)
II	38 (35.5)	176 (24)
III	42 (39.8)	87 (12)
R‐ISS (*n*, %)		
I	18/80 evaluable pts (22.5)	N/A
II	26/80 evaluable pts (32.5)	N/A
III	36/80 evaluable pts (45.0)	N/A
R2‐ISS (*n*, %)		
I	17/72 evaluable pts (23.6)	N/A
II	13/72 evaluable pts (18.0)	N/A
III	34/72 evaluable pts (47.2)	N/A
IV	8/72 evaluable pts (11.2)	N/A
Cytogenetic risk (*n*, %)		(Data not available in 24%)
Standard risk	34/75 evaluable pts (45.3)	415 (57)
High risk	41/75 evaluable pts (54.7)	137 (19)
del17p	19/75 evaluable pts (25.3)	N/A
Creatinine clearance (*n*, %)		
eGFR >60 mL/min	81 (68.4)	542 (73)
eGRR <60 mL/min	25 (31.6)	169 (23) (eGFR 30 to ≤60 mL/min)
Prior lines of treatment (*n*, %)		
1	42 (39.8)	411 (61)
2	23 (21.7)	208 (29)
≥3	41 (38.5)	73 (10) (3 prior lines)
Prior lenalidomide (*n*, %)	61 (57.5)	88/722 (12)
Lenalidomide‐exposed, non‐refractory	39 (36.8)	88/722 (12)
Lenalidomide‐refractory	22 (20.7)	0
Lenalidomide immediately prior to IRd (*n*, %)	16 (15.5)	N/A
Lenalidomide‐exposed, non‐refractory	9 (8.7)	N/A
Lenalidomide‐refractory	7 (6.8)	0
Prior bortezomib (*n*, %)	83 (78.3)	498 (69)
Bortezomib‐exposed, non‐refractory	69 (65.1)	486 (67)
Bortezomib‐refractory	14 (13.2)	12 (2)
Prior ASCT	44.5 (42)	411 (57)

*Note*: High cytogenetic risk defined as the presence of [del17p and/or t(4;14) and/or t(14;16) and/or 1 g gain/amp], detected by fluorescence in situ hybridization (FISH).

Abbreviations: ASCT, autologous stem cell transplant; ECOG, Eastern Cooperative Oncology Group; Egfr EGFR, estimated glomerular filtration rate; ISS, International Staging System; *n*, number; N/A, not available; R‐ISS, Revised International Staging System; R2‐ISS, Second Revision of the International Staging System.

### 3.2 Treatment received

For just over half of the pts (50.8%) the Len starting dose was 15 mg or less according to standard recommendations for cytopenia, RI and age. Ixa and Dex were started according to the planned schedule in all patients. Antithrombotic prophylaxis was adopted in all pts except 3, mainly aspirin or heparin (both in 28.3% of pts).

### 3.3 Safety

The most frequent toxicity was hematological, mainly grades (G) 1 and 2, with 38.7%, 36.8% and 34.9% of anemia, thrombocytopenia, and neutropenia, respectively.

Overall, G ≥3 hematological AEs were reported in 26.4% of pts. More specifically, G ≥3 anemia occurred in 4.7%, thrombocytopenia in 16%, neutropenia in 12.3%. Infections, GI toxicity (mainly diarrhea), skin rash and peripheral neuropathy (PN) were the commonest non‐hematological toxicities of any grade. G ≥3 non‐hematologic AEs overall occurred in 24.5% of pts and included mostly infections (9.4%) and GI toxicity (8.5%, including diarrhea in 5.7% and hepatotoxicity in 2.8%). G ≥3 VTE, skin rash and weakness were reported in 2.8%, 1.9%, and 1.9% respectively. Notably, the incidence of any grade PN was 26.4%; however, no new onset G ≥3 neurological AE was observed. When analyzing pts aged ≥75, the rates of any grade and G ≥3 hematological and non‐hematological AEs were similar to the overall population, except for a higher incidence of G ≥3 thrombocytopenia (22.2%) and skin rash (25% any grade, 5.5% G ≥3) (Table 2).

**TABLE 2:** Adverse events (all grades and grade ≥3).

Adverse event	*n* of pts (%)—Overall population		*n* of pts (%)—Pts aged ≥75 (*n* = 36)	
	All grades	Grade ≥3	All grades	Grade ≥3
Hematological				
Anemia	41 (38.7)	5 (4.7)	12 (33.3)	1 (2.8)
Thrombocytopenia	39 (36.8)	18 (16)	14 (38.9)	8 (22.2)
Neutropenia	37 (34.9)	13 (12.3)	14 (38.9)	4 (11.1)
Non‐hematological				
Infection	23 (21.7)	10 (9.4)	5 (13.9)	2 (5.5)
GI (diarrhea)	31 (29.2)	6 (5.7)	10 (27.8)	3 (8.3)
GI (LFTs abnormalities)	7 (6.6)	3 (2.8)	1 (2.8)	0
VTE	4 (3.8)	3 (2.8)	0	0
Skin rash	15 (14.1)	2 (1.9)	9 (25)	2 (5.5)
Peripheral neuropathy	28 (26.4)	0	6 (16.7)	0
Other	17 (16.0)	2 (1.9)	3 (8.3)	0

Abbreviations: GI, gastrointestinal; LFTs, liver function tests; n, number; VTE, venous thromboembolism.

As previously mentioned, only 13.2% of the pts discontinued treatment for toxicity (2.8% due to G ≥3 hematological AEs, 10.4% due to non‐hematological AEs, mainly infections). Overall, in 12.3% of pts a dose reduction of one or more drugs occurred for G ≥3 AEs. When specifically considering hematological toxicity, 6.6% and 4.7% of pts needed Len and Ixa dose reduction, respectively, while only 2.8% required reduction of both drugs. With regard to non‐hematological toxicity, 8.5% and 5.7% needed Len and Ixa dose reduction, respectively, mainly due to diarrhea (4.7% and 3.8%). 4.7% required reduction of both drugs, in all cases due to diarrhea.

In pts aged ≥75 at the time of IRd initiation, the rate of treatment discontinuation due to G ≥3 hematological AEs was slightly higher (5.5%), while no differences were noted in terms of discontinuation for non‐hematological AEs and dose reductions, both for Len and Ixa (Table 3). Notably, among pts aged ≥75, 48% received a reduced Len starting dose (5–15 mg), which is comparable to the overall population (50.8%).

**TABLE 3:** Incidence of dose adjustments and drug discontinuation.

	*n* of pts (%) – Overall population (*n* = 106)	*n* of pts (%) – Pts aged ≥75 (*n* = 36)
Discontinuation of treatment due to hematological AEs	3 (2.8)	2 (5.5)
Discontinuation of treatment due to non‐hematological AEs	11 (10.4) (infection, VTE, LFTs abnormalities, weakness, rash, diarrhea)	3 (8.3) (rash, diarrhea, infection)
Dose reduction in lenalidomide due to hematological AEs	7 (6.6)	3 (8.3)
Dose reduction in lenalidomide due to non‐hematological AEs	9 (8.5)	3 (8.3)
Dose reduction in ixazomib due to hematological AEs	5 (4.7)	1 (2.8)
Dose reduction in ixazomib due to non‐hematological AEs	6 (5.7)	1 (2.8)

Abbreviations: AEs, adverse events; LFTs, liver function tests; *n*, number; pts, patients; VTE, venous thromboembolism.

### 3.4 Efficacy and survival outcomes

**FIGURE 1:**
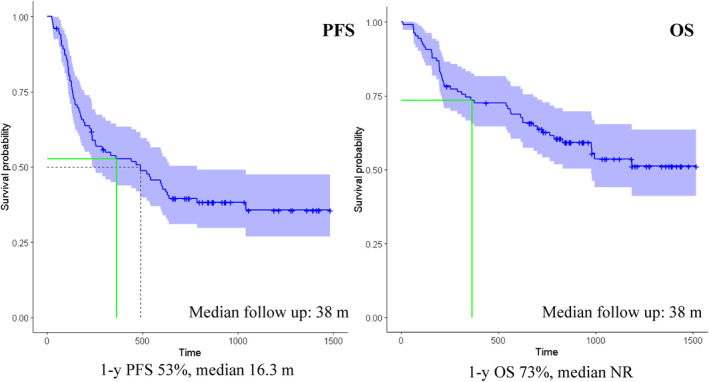
Kaplan–Meier curves of PFS (A) and OS (B) of the overall study population.

Treatment responses are reported in Table 4. The ORR was 56.4%, including 30%≥ VGPR and 11.2% CR. Among pts who were Len‐exposed, non‐refractory, the ORR was similar to the overall population (54%) although with a lower rate of best response ≥VGPR (25.6%). Len‐refractoriness impacted negatively both on ORR (36.4%) and rate of ≥VGPR (27.3%). At the time of analysis, 18.9% of pts were on ongoing treatment, 81.1% had discontinued IRd treatment, mainly (59.4%) for PD, and in a lower proportion (13.2%) for toxicity. By the cutoff date, 43.3% pts had died, mostly due to PD (28.3%); in 10.4% the death was secondary to toxicity, in almost all cases infections. In 2 pts, the cause of death was pulmonary embolism and myocardial infarction, respectively. With a median follow‐up of 38 m, the mPFS was 16 m, mOS not reached (NR), and the 1‐year (1‐y) OS rate 73% (Figure 1).

**TABLE 4:** Treatment responses.

Overall response rate, overall population (*n* = 106) (*n*, %)	64 (56.4)
Best response, overall population (*n*, %)	
CR	12 (11.2)
VGPR	20 (18.8)
VGPR/CR	32 (30)
PR	28 (26.4)
MR/SD	38 (35.8)
PD	8 (7.5)
Overall response rate in Len‐exposed pts (*n* = 61) (*n*, %)	29 (47.5)
Best response VGPR/CR	16 (26.2)
Overall response rate in Len‐exposed, non‐refractory pts (*n* = 39) (*n*, %)	21 (54)
Best response VGPR/CR	10/39 (25.6)
Overall response rate in Len‐refractory pts (*n* = 22) (*n*, %)	8/22 (36.4)
Best response VGPR/CR	6/22 (27.3)

Abbreviations: CR, complete response; Len, lenalidomide; MR, minimal response; *n*, number; PD, progressive disease; PR, partial response; pts, patients; SD, stable disease; VGPR, very good partial response.

Aiming to identify prognostic factors affecting PFS, Cox regression univariate and multivariate analyses were performed (Table 5). By univariate subgroup analysis, an extended PFS was observed for pts who achieved ≥VGPR as best response (21.2 vs. 7.8 m, HR = 0.55, 95% CI: 0.31–0.99, *p* 0.04), with prolonged time (≥5 years) from diagnosis to IRd (26.2 vs. 7.6 m, HR = 0.48, 95% CI: 0.29–0.79, *p* 0.004), who had received 1 pLoT when compared to the rest of the population, although with weak statistical significance (34.4 vs. 15.5 m, HR = 0.59, 95% CI: 0.33–1.02, *p* 0.07), without previous exposure to Len (Len‐naïve) compared with Len‐exposed (mPFS NR vs. 8.4 m, 95% CI: 6.4–20.2, *p* = 0.04). Interestingly, however, in pts who had been previously exposed, but were not refractory to Len, mPFS was comparable to the overall population (16 m). Conversely, refractoriness to Len has a strong negative prognostic impact as it is associated with a significantly worse PFS compared with Len‐naïve (4.6 vs. NA, HR = 2.45, 95% CI: 1.27–4.71, *p* = 0.007). Pts who had been exposed but were not refractory to a Len‐based regimen immediately prior to IRd, had a mPFS similar to Len‐naïve (mPFS 17.4 m in Len‐naïve, 18 m in Len‐exposed, non‐refractory, 4.2 m in Len‐refractory immediately prior to IRd). Bortezomib‐refractory pts had a shorter PFS compared with pts who had exposed but, but were not refractory to Bortezomib (4.3 vs. 18 m, HR = 2.23, 95% CI: 1.13–4.41, *p* = 0.02). Prolonged mPFS was observed in pts aged ≥70 vs. <70 (20 vs. 7.7 m, HR = 0.56, 95% CI: 0.34–0.93, *p* = 0.02), particularly in the range between 70 and 74 compared with the younger age group (26 vs. 16 m vs. 7.4 in the age groups ≥70–74, ≥75 and <70 respectively, *p* = 0.04). The baseline characteristics of pts aged >70 were similar to the overall population except for a lower incidence of prior ASCT (21.8% vs. 42.4%). Among pts aged ≥70, the proportion with only 1 pLoT (33.3%), exposure (55%) and refractoriness to prior Len (18.8%) were in line with the overall population.

**TABLE 5:** Univariate and multivariate analyses of variables affecting PFS.

Independent variable	Cox proportional hazard regression analysis for PFS						
		Univariate			Multivariate		
		HR	95% CI	*p* value	HR	95% CI	*p* value
Time from diagnosis to IRd	≥5 y versus <5 y	0.48	0.29–0.79	0.004	0.32	0.18–0.57	0.0001
Age	≥70 versus <70	0.56	0.34–0.93	0.02	0.6	0.36–1.002	0.05
Best response	≥VGPR versus <VGPR	0.55	0.31–0.99	0.04	0.67	0.36–1.24	0.20
Len‐exposure status	Exposed, non‐refractory to Len non immediately prior to IRd versus Len‐naïve	1.62	0.87–3.03	0.13	2.14	1.07–4.27	0.03
	Refractory to Len non immediately prior to IRd versus Len‐naïve	2.13	1.02–4.42	0.04	3.33	1.46–7.61	0.004
	Exposed, non‐refractory to Len immediately prior to IRd versus Len‐naïve	1.18	0.44–3.19	0.74	2.04	0.69–6.02	0.19
	Refractory to Len immediately prior to IRd versus Len‐naïve	3.59	1.41–9.16	0.007	4.31	1.56–11.94	0.004
Bor‐exposure status	Refractory to Bor versus Bor‐exposed, non‐refractory	2.23	1.13–4.41	0.02	—		
	Bor‐naïve versus Bor‐exposed, non‐refractory	1.19	0.53–2.67	0.67	—		
Prior lines of therapy	1 versus ≥2	0.59	0.33–1.02	0.07	—		
Cytogenetics	HR versus SR	1.33	0.74–2.4	0.33	—		
Del17p	del17p versus no del17p	0.97	0.48–1.93	0.93	—		
Starting Len dose	25 versus <25 mg	1.29	0.69–2.39	0.4	—		
Time interval between the last Len administration and IRd	≥12 versus <12 m	0.98	0.52–1.85	0.97	—		
eGFR	>60 versus ≤60 mL/min	0.77	0.43–1.39	0.4	—		

Abbreviations: Bor, bortezomib; HR, high risk; Len, lenalidomide; m, months; SR, standard risk; VGPR, very good partial response; y, years.

The presence of RI (eGRF ≤60 mL/min) may be associated with a trend toward reduced PFS (17 vs. 8.5 m, *p* = 0.4). mPFS was higher in pts with standard cytogenetic risk compared to those with high risk (21 vs. 11 m); however, the difference was not statistically significant (*p* = 0.3). When we specifically analyzed the impact of del17p, no statistically significant difference emerged in terms of mPFS for pts with or without del17p (14.7 vs. 17.4 m, *p* = 0.9). Furthermore, no significant difference in PFS was found based on the time interval (≥12 vs. <12 m) between the last Len administration and IRd (8.2 vs. 7.7, *p* = 1); starting Len dose 25 mg versus <25 mg (8 vs. 16.2 m, *p* = 0.4); ISS I‐II versus III (16.2 vs. 14.7 m, *p* = 1).

**FIGURE 2:**
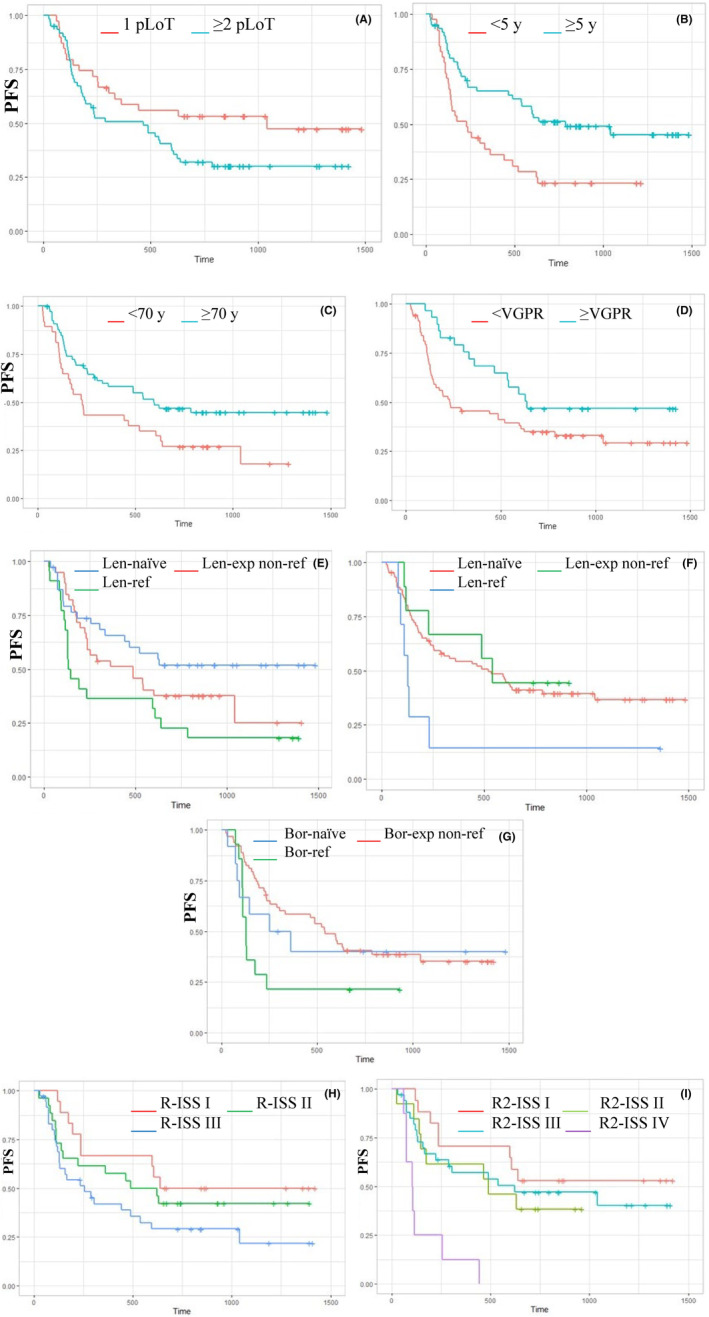
Kaplan–Meier curves of PFS based on the number of pLoT (A), time from diagnosis (B), age (C), best response (D), exposure/refractoriness to Len in any prior line (E) and immediately prior to IRd (F), exposure/refractoriness to prior bortezomib (G), R‐ISS (H), R2‐ISS (I). Bor‐exp,bortezomib‐exposed; Len‐exp,lenalidomide‐exposed; NR,not reached; pLoT,prior lines of therapy; R2‐ISS, second revision of the International Staging System; R‐ISS,Revised International Staging System; VGPR,very good partial response; y,years.

The standard‐of‐care risk stratification model is currently represented by the Revised International Staging System (R‐ISS), incorporating two further prognostic factors into ISS: cytogenetic risk as assessed by FISH and LDH level.^22^ The second revision of the International Staging System (R2‐ISS) analyzes the additive value of each single risk feature, including chromosome 1q gain/amplification (1q+) that recently proved to be a poor prognostic factor.^23^ In this real‐world, RRMM setting, however, information on cytogenetics by FISH, and, more specifically, on 1q+, and LDH levels is not available in a relevant proportion of pts, limiting the subgroup analyses based on R‐ISS and R2‐ISS to 80 pts and 72 pts, respectively. By univariate subgroup analysis, a statistically significant difference in PFS emerged for pts in stage R‐ISS III compared to R‐ISS I (*p* = 0.05) and R2‐ISS IV compared with R2‐ISS I (*p* = 0.0001). However, the low number of cases did not allow us to analyze the independent prognostic value of these parameters in a multivariate analysis. The Kaplan–Meier curves of PFS for the above‐mentioned factors are reported in Figure 2.

A survival benefit emerged in pts achieving ≥VGPR compared to pts achieving less than VGPR as best response (1‐y OS 82.4% vs. 68.1%, *p* = 0.02). Conversely, pts achieving less than PR had only 50% 1‐y OS. A 1‐y OS advantage was also observed in pts with prolonged time (≥5 years) from diagnosis to IRd (80.9% vs. 61.9%, *p* = 0.07), no refractoriness to prior Len‐based therapy (79.5% vs. 52.2%, *p* = 0.1), absence of HRCA (76.5% vs. 65.5%, *p* = 0.1). Interestingly, the 1 y‐OS in Len‐exposed, non‐refractory pts was similar to naïve pts (80%); similarly, pts exposed, non‐refractory to a Len‐based regimen immediately prior to IRd had 100% 1‐y OS, in the face of 50% in pts refractory to Len as immediately prior to IRd. Extended OS was associated with high quality responses (≥VGPR) compared with pts achieving <VGPR (mOS NR vs. 39.5 vs. 20.8 m in pts achieving ≥VGPR, PR, and <PR respectively, *p* = 0.002), age 70–74 (mOS NR vs. 32.6 vs. 39.5 m in the age groups 70–74, ≥75, and <70 respectively, *p* = 0.05), and time ≥5 years from diagnosis to IRd (mPFS NR vs. 25.5 m, *p* = 0.07).

**FIGURE 3:**
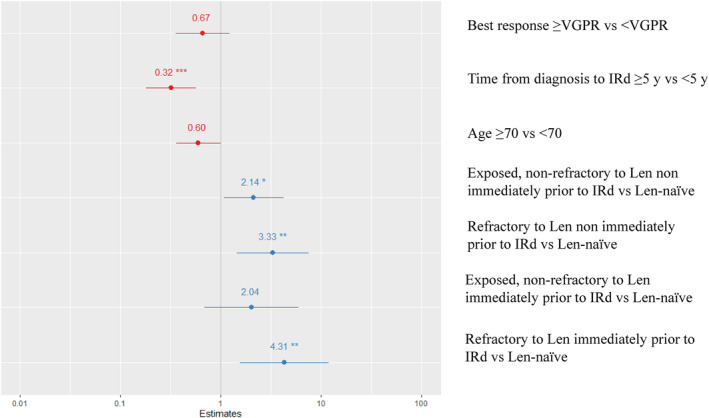
Multivariate analysis of variables affecting PFS. len,lenalidomide; VGPR,very good partial response; y,years. ^***^ p<0.001; ^**^ p<0.01; ^*^ p<0.05.

All the variables that showed a significant prognostic value (*p* = <0.05) in the univariate analysis were used in a multivariate analysis (Table 5, Figure 3). By multivariate Cox regression analysis, the only independent predictors of PFS were age ≥70 (HR = 0.6, 95% CI: 0.36–1.002, *p* = 0.05), time from diagnosis ≥5 years (HR = 0.32, 95% CI: 0.18–0.57, *p* = 0.0001), and refractoriness to Len, both immediately prior (HR = 4.31, 95% CI 1.56–11.94, *p* = 0.004) and non‐immediately prior to IRd (HR = 3.33, 95% CI 1.46–7.61, *p* = 0.004) compared with Len‐naïve. Len‐exposure non immediately prior to IRd has a weak statistical significance as an independent negative predictor (HR = 2.14, 95% CI 1.07–4.27, *p* = 0.03).

## 4 DISCUSSION

The global phase 3 TOURMALINE‐MM1 study, including 722 pts with RRMM who had received 1–3 previous lines of therapy, showed a significant improvement of PFS in pts treated with the combination of IRd compared with Rd (mPFS 20.6 vs. 14.7 m, HR = 0.74, *p* = 0.012).^1^ The FDA approval of Ixa for use in combination with Len and Dex for pts who have received at least 1 pLoT, based on the results of the pivotal study, allowed practitioners to provide RRMM pts an all‐oral triplet therapy. Nevertheless, the baseline characteristics of the study population in the MM1 study only partially reflect those in the real‐world population treated with IRd. The study population in the MM1 study included a small proportion of pts aged ≥75 (15%, median age 66), with impaired PS (ECOG 2 6%), advanced stage disease (ISS III 12%), moderate RI, defined as creatinine clearance 30 to <60 mL/min per 1.73 m^2^ (23%), 3 pLoT (10%), Len‐exposure (12%), HRCA (19%) (Table 1). In the routine clinical practice, the all‐oral route of administration and the favorable safety profile make this regimen potentially suitable for the treatment of elderly pts with comorbidities and/or impaired PS.^5^, ^6^ Several observational studies have indeed reported a higher prevalence of older age, impaired PS, advanced stage disease, >2 pLoT in the real‐world population treated with IRd, showing comparable effectiveness with OR rates ranging from 60% to 74% and mPFS ranging from 11.4 to 43 m.^7^, ^8^, ^9^, ^10^, ^11^, ^12^, ^13^, ^14^, ^15^ Moreover, a significant proportion of pts (17%–39%) in the real‐world setting has previous exposure to Len compared to only 12% in the MM1 study. Furthermore, Len‐refractory pts were excluded from the pivotal trial and are very poorly represented in the real‐world published data. However, an increasing proportion of MM pts, both transplant eligible and non‐transplant eligible, receive Len‐based first line regimens until progression.^24^, ^25^, ^26^ As a result, the vast majority are Len‐exposed and, more often, Len‐refractory as early as at first relapse, raising the issue of the optimal placing of this Len‐based triplet.^16^, ^17^

We therefore conducted a retrospective/prospective analysis to assess the efficacy and safety of IRd in a real‐world population of 106 patients treated in 21 centers in the North of Italy between January 2017 and May 2021.

Older age (≥75), advanced ISS stage and HRC disease, poor PS, impaired renal function, heavily pre‐treated, Lenalidomide‐exposed, and refractory pts were highly represented in the study population. In terms of efficacy, the ORR (56.4%) and rate of deep responses (≥VGPR 30%) reported in the present study were inferior to those in the TOURMALINE‐MM1 (ORR 78%, ≥VGPR 48%), possibly as a result of the unfavorable characteristics of this study population in terms of prevalence of frailty, high‐risk disease and previous treatment exposures. Consistently, mPFS (16.3 m) and 1‐y OS (73%) were shorter compared with the TOURMALINE‐MM1.

In line with previously published real‐world studies of IRd,^9^, ^10^, ^15^ extended PFS emerged in pts with fewer pLoT. mPFS in pts with only 1 pLoT (34.4 m) is approximately twofold longer than with ≥2 pLoT, although with weak statistical significance, supporting the early use of IRd as an effective salvage therapy. It should be noted that in Italy Ixa is reimbursed in pts with MM who received at least 2 pLoT, or in pts with HRCA after only 1 pLoT.^27^ It follows that all the pts treated with IRd at first relapse in this study had HRCA, and that early treatment may be associated with a partial overcoming of the negative prognostic impact of cytogenetics.

In the present analysis, Len exposure (57.5% of pts, including both Len‐sensitive and refractory—36.8% and 20.7%, respectively), negatively impacted on PFS compared to no exposure, in line with previously published real‐world data.^9^ In contrast, recently published data of a large, non‐interventional prospective study on 376 pts, showed that among pts receiving IRd after 1 or 2 pLoT, mPFS was similar for pts previously exposed to Len (19.5 m) than for those Len‐naïve (22.6 m, *p* = 0.29).^15^ It should be noted, however, that in the present study the analysis of the impact of prior Len exposure was performed in a population also including pts with >2 pLoTs and a high proportion of Len‐refractory pts. Interestingly, mPFS in previously exposed, non‐refractory pts was comparable to the overall population (16 m). When considering pts exposed, non‐refractory to a Len‐based regimen immediately prior to IRd, mPFS (18 m) was comparable to Len‐naïve pts (17.4 m). Exposure with no refractoriness to Len immediately prior to IRd did not prove statistically significant as an independent negative predictor compared to non‐exposure. Conversely, refractoriness to Len has the strongest negative impact on PFS (4.2 and 4.6 m with Len immediately prior and non‐immediately prior to IRd, respectively) and 1‐y OS (52.2% vs. 79.5% in Len‐ refractory and non‐refractory pts), and proved to be a strong independent negative predictor in terms of PFS both immediately prior (HR 4.31) and non‐immediately prior to IRd (HR = 3.33) compared to Len‐naïve. These data support the potential efficacy of IRd in pts with suboptimal response to a prior Len‐based regimen and in those who have discontinued Len for reasons other than progressive disease and argue against the use of IRd in pts who have clearly proved to be refractory to Len, regardless of the timing of administration.

By contrast, the interval between the last Len administration and IRd and the Len starting dose (25 vs. <25 mg) did not impact on PFS. By univariate analysis, Bortezomib‐refractory pts had a shorter PFS compared with pts who had been exposed but were not refractory to bortezomib (4.3 vs. 18 m, HR = 2.23, 95% CI: 1.13–4.41, *p* = 0.02), suggesting that pts who are resistant to bortezomib might be, at least partially, cross‐resistant to the second‐generation, oral proteasome inhibitor Ixa.

No statistically significant difference in outcome emerged in pts with HRCA, and more specifically with del17p, compared with standard cytogenetic risk. Therefore, our real‐world data are consistent with those from TOURMALINE‐MM1 with regard to the ability of this triplet regimen to overcome, at least partially, the negative impact of HRCA, including del17p. These results should be interpreted with caution since cut‐off thresholds to define FISH positivity were not set. As previously mentioned, all the pts treated with IRd at first relapse in this study had HRCA, and early treatment may have affected favorably the outcome of HRC pts treated with IRd.

Improved PFS and 1‐y OS outcomes were associated with high‐quality responses (≥VGPR) and time from diagnosis to IRd ≥5 years. A prolonged time from diagnosis may reflect an indolent course of the disease, affecting positively the PFS on the previous line(s) of therapy, and emerged as an independent positive predictor (HR 0.32) in multivariate analysis. Consistently, previous data have shown a positive correlation of PFS of induction and PFS in RRMM pts treated with IRd.^8^

As for the impact of age, extended PFS was reported in pts aged ≥70 compared with the younger age group. The PFS benefit translated into a survival advantage in the age group 70–74 compared with the rest of the population. As pointed out earlier, the baseline characteristics of pts aged ≥70 were similar to the overall population with the exception of a lower incidence of prior ASCT (21.8% vs. 42.4%). Age ≥70 maintained an independent role as a positive prognostic factor in the multivariate analysis (HR 0.6). Consistently, data recently reported by the French group did not report significant differences in terms of PFS and ORR in pts younger and older than 80 (mPFS 19.1 vs. 17.4 m, *p* = 0.06; ORR 72.4% and 76.8% respectively).^15^ Unlike age, frailty has been reported to affect negatively the outcome of pts treated with IRd in real life.^15^ In the present study, no specific assessment of frailty has been performed.

The safety profile is favorable, with no new safety signals observed, in elderly pts as well as in the overall population. The rates of G ≥3 hematological and non‐hematological toxicities, treatment discontinuation and dose reduction of one or both of the drugs were low and comparable in pts aged ≥75 and the overall population. In the newly diagnosed MM setting, the prospective, phase 4 US MM‐6 study showed that In‐Class Transition from parenteral bortezomib‐based induction to all‐oral IRd therapy allowed long‐term PI‐based treatment with improved responses in both pts aged <75 and ≥75. Similarly to our findings, the safety profile was favorable with rates of treatment‐emergent AEs and AEs leading to discontinuation comparable in both age groups and no adverse impact on quality of life.^28^ Our data, in accordance with the previously published data, support the interest of IRd in an older population, with acceptable tolerance and effectiveness at least comparable to a younger population.

Our analysis presents some limitations. Firstly, the retrospective nature of the study. Furthermore, in this real‐world, RRMM setting, the cytogenetic profile was available in 70% of pts, and just over half (57%) were, more specifically, evaluable for 1q+, resulting in the inability to perform subgroup analyses based on R‐ISS and R2‐ISS. The cutoff thresholds to define FISH positivity, including del17p, were not set. This might, at least partially, explain the high incidence of HRCA in this study population compared to other published series and suggests caution in the interpretation of the results with respect to cytogenetics. Moreover, the exact timing and extent of Len, Ixa and Dex dose modifications were not systematically reported in the retrospective analysis, preventing us from assessing the impact of dose intensity on outcomes. Another limitation is the lack of MRD evaluation in pts achieving VGPR/CR.

## 5 CONCLUSION

In conclusion, in a real‐life population with adverse prognostic characteristics in terms of older age, PS, cytogenetic risk, prior exposure and refractoriness to Len, IRd demonstrated inferior ORR, rates of ≥VGPR and PFS as compared to the TOURMALINE‐MM1, with a favorable safety profile and no new safety concerns. Prolonged time from diagnosis to IRd and age ≥70 were independently associated with favorable outcomes. While refractoriness to prior Len has proved to be a strong independent negative prognostic factor in pts treated with IRd, regardless of timing of administration, our data support the potential efficacy of IRd in pts who were Len‐sensitive or had a suboptimal response to a prior Len‐based regimen. Moreover, treatment with IRd in earlier lines of therapy might overcome the adverse impact of high‐risk cytogenetics. IRd might, therefore, represent an effective and safe combination in selected RRMM pts with an indolent disease course and slow relapse kinetics, in early lines of treatment, who are Len‐sensitive, independent of age, and cytogenetic risk.

## AUTHOR CONTRIBUTIONS

**Anna Furlan:** Conceptualization (lead); data curation (lead); investigation (equal); methodology (lead); writing – original draft (lead). **Michele Cea:** Investigation (equal). **Laura Pavan:** Investigation (equal). **Monica Galli:** Investigation (equal). **Cristina Clissa:** Investigation (equal). **Silvia Mangiacavalli:** Investigation (equal). **Anna Maria Cafro:** Investigation (equal). **Stefania Girlanda:** Investigation (equal). **Francesca Patriarca:** Investigation (equal). **Claudia Minotto:** Investigation (equal). **Giovanni Bertoldero:** Investigation (equal). **Gregorio Barilà:** Investigation (equal). **Anna Pascarella:** Investigation (equal). **Albana Lico:** Investigation (equal). **Rossella Paolini:** Investigation (equal). **Nicholas Rabassi:** Investigation (equal). **Norbert Pescosta:** Investigation (equal). **Marika Porrazzo:** Investigation (equal). **Giovanni De Sabbata:** Investigation (equal). **Alessandra Pompa:** Investigation (equal). **Giulia Bega:** Investigation (equal). **Stefania Cavallin:** Investigation (equal). **Francesca Guidotti:** Investigation (equal). **Magda Marcatti:** Investigation (equal). **Maurizio Rupolo:** Investigation (equal). **Angelo Belotti:** Investigation (equal). **Filippo Gherlinzoni:** Investigation (equal). **Renato Zambello:** Investigation (equal); supervision (lead); writing – review and editing (lead).

## CONFLICT OF INTEREST STATEMENT

The authors declare that they have no competing interests.

## FUNDING INFORMATION

This research did not receive any specific grant from funding agencies in the public, commercial, or not‐for‐profit sectors.

## ETHICS STATEMENT

All procedures performed in studies involving human participants were in accordance with the ethical standards of the institutional and/or national research committee and with the 1964 Helsinki Declaration and its later amendments or comparable ethical standards. The study was approved by the institutional Ethical Committee (Ethical Committee for Clinical Experimentation, province of Treviso and Belluno, Veneto Region).

## INFORMED CONSENT

Informed consent was obtained from all individual participants included in the study.

## Data Availability

The data that support the findings of this study are available from the corresponding author upon reasonable request.
